# A Spatiotemporal Probabilistic Graphical Model Based on Adaptive Expectation-Maximization Attention for Individual Trajectory Reconstruction Considering Incomplete Observations

**DOI:** 10.3390/e26050388

**Published:** 2024-04-30

**Authors:** Xuan Sun, Jianyuan Guo, Yong Qin, Xuanchuan Zheng, Shifeng Xiong, Jie He, Qi Sun, Limin Jia

**Affiliations:** 1 School of Traffic and Transportation, Beijing Jiaotong University, No. 3 Shangyuancun, Haidian District, Beijing 100044, China; xuansun@bjtu.edu.cn (X.S.); 20114089@bjtu.edu.cn (X.Z.); 23120804@bjtu.edu.cn (J.H.); 2 State Key Laboratory of Advanced Rail Autonomous Operation, Beijing Jiaotong University, No. 3 Shangyuancun, Haidian District, Beijing 100044, China; lmjia@bjtu.edu.cn; 3 Beijing Urban Construction Design & Development Group Co., Ltd., No. 5 Fuchengmen North Street, Xicheng District, Beijing 100032, China; 4 NCMIS, KLSC, Academy of Mathematics and Systems Science, Chinese Academy of Sciences, Beijing 100190, China; xiong@amss.ac.cn; 5 Beijing Metro Network Administration Co., Ltd., No. 6 Xiaoying North Road, Chaoyang District, Beijing 100020, China; sunqi@bmncc.com.cn

**Keywords:** urban rail transit, trajectory prediction, probabilistic graphical model, expectation-maximization algorithm, attention mechanism

## Abstract

Spatiotemporal information on individual trajectories in urban rail transit is important for operational strategy adjustment, personalized recommendation, and emergency command decision-making. However, due to the lack of journey observations, it is difficult to accurately infer unknown information from trajectories based only on AFC and AVL data. To address the problem, this paper proposes a spatiotemporal probabilistic graphical model based on adaptive expectation maximization attention (STPGM-AEMA) to achieve the reconstruction of individual trajectories. The approach consists of three steps: first, the potential train alternative set and the egress time alternative set of individuals are obtained through data mining and combinatorial enumeration. Then, global and local potential variables are introduced to construct a spatiotemporal probabilistic graphical model, provide the inference process for unknown events, and state information about individual trajectories. Further, considering the effect of missing data, an attention mechanism-enhanced expectation-maximization algorithm is proposed to achieve maximum likelihood estimation of individual trajectories. Finally, typical datasets of origin-destination pairs and actual individual trajectory tracking data are used to validate the effectiveness of the proposed method. The results show that the STPGM-AEMA method is more than 95% accurate in recovering missing information in the observed data, which is at least 15% more accurate than the traditional methods (i.e., PTAM-MLE and MPTAM-EM).

## 1. Introduction

Currently, urban rail transit (URT) has become the preferred public transport mode for residents due to its large capacity and high efficiency. For example, in Beijing, the total number of passengers reached 5.327 billion in 2022, of which 42.5% were transported by URT [1]. Due to the large proportion of transportation, the URT system also faces many problems, such as the fact that it is often difficult to transport passengers in a timely manner during peak traffic hours, which leads to crowding induced by passengers waiting for trains on platforms and other areas [2,3,4]. Furthermore, factors such as the capacity of different train types and station layouts also result in uncertain waiting times and complicated travel choices for passengers [2,5,6,7,8,9,10,11].

To better monitor the URT system’s status and optimize train scheduling, precise access to spatiotemporal characteristics and semantic information of passengers is a prerequisite [2,4,5,10,12,13,14,15,16]. The prediction of individual mobility using data-driven modeling approaches based on Automatic Fare Collection (AFC) data and Automatic Vehicle Location (AVL) data has been a hot research topic in recent years. Meanwhile, individual movement information can also be used for emergency commands or providing personalized recommendation services [10]. Among the studies on individual mobility modeling in urban rail transit, scholars mainly carry out three aspects to achieve accurate prediction of individual movement, namely, travel pattern mining [17,18], route choice model [19,20,21,22,23,24], and individual trajectory inference [10,15,25,26,27]. These studies are categorized into network-level, path-level, and train-level according to the scale of the URT system.

In the first aspect of research, unsupervised learning methods (e.g., K-means, LDA) are used to mine job-housing relationships or travel patterns about passengers, which can subdivide passengers into different groups [5,18,28,29,30,31,32]. For example, Cheng et al. [18] developed a topic model to predict passengers’ travel destinations, thereby distinguishing between commuters and non-commuters. However, the focus of these methods is generally to construct input features (such as travel days, travel time, etc.), which are primarily used to support macro-level transportation planning or the prediction of new lines, offering limited assistance for operational-level adjustments and strategies [32].

Furthermore, considering the path-level, passengers need to be matched to one physical path between Origin-Destination (OD) pairs. Thus, large research on route choice and assignment models consists of three main methodologies: the Logit model based on labeled data, the clustering model based on unsupervised learning, and the probability-based generative model [3,21,33,34,35,36]. The Logit model and its variants are generally established by considering the number of transfers, distance, waiting time, etc. [6,34,37,38]. Some scholars have adopted unsupervised clustering methods for exploration, e.g., Fu et al. [39] combined the AFC data of London Underground with the Gaussian Mixture Model (GMM) within a Naive Bayesian framework to calculate the line selection probability. Wu et al. [36] proposed a fuzzy matching method to assign the passenger flow to each line using the AFC data. Probabilistic generative models appeared almost simultaneously with clustering methods [40]. They are mainly based on Bayes’ rule or frequency-based statistical inference methods. Sun et al. [21] proposed a comprehensive Bayesian inference framework that is combined with the Metropolis–Hastings (M-H) algorithm to provide a posterior distribution for route choice. From an application perspective, at the path-level, these researchers are still unable to obtain fine-grained information about individual trajectories and face methodological limitations such as poor stability or over-reliance on survey data. 

Moreover, some scholars have expanded individual trajectory reconstruction (ITR) from the path-level to the train-level by integrating AFC data with other data, focusing on models for matching passengers to the train. Current research primarily rely on the Rule-based Method (RM) and the Probabilistic Generative Model (PGM). RM directly utilizes the segmentation and concatenation of AFC and AVL data to mine the matching relationship between passengers and trains [15,26]. However, the spatiotemporal constraints in such methods are considered hard constraints, lacking detailed depictions of passenger behaviors. Studies based on PGM refine the modeling of passengers’ left-behind or waiting behaviors at stations [2,12,20,41,42,43], such as PTAM [20] and LBPMF [42]. However, some essential parameters in these studies still need manual surveys (walking speed, etc.). The improved MPTAM model established by Xiong et al. [12] can automatically fit parameters without resorting to external data. Considering the randomness of boarding choice at the individual level, the error of these researches may be large. Further exploring the inherent value of data to replace manual surveys presents a worthwhile approach for obtaining passengers’ spatiotemporal trajectories to explore.

In summary, the existing methods have the problems of the high cost of manual investigation, large sample randomness, coarse sampling granularity, etc. Therefore, it is extremely challenging to fully explore the hidden information and obtain the unknown state and semantic information (e.g., waiting time, walking time, etc.) of each passenger without relying on any manual investigation.

In this paper, a spatiotemporal probabilistic graphical model based on the adaptive expectation-maximization attention algorithm (STPGM-AEMA) is proposed. The method can effectively recover the rich semantic and state information of each individual trajectory only from Automatic Fare Collection (AFC) data and Automatic Vehicle Location (AVL) data. Specifically, the main contributions of the paper are as follows:

1. A spatiotemporal probabilistic graphical model (STPGM) is proposed with global and local interactive representation to capture the complex spatiotemporal dependencies between individuals and system components (stations or trains) and obtain the individual trajectory at the train level, operating without manual survey data input.
2. Considering the sensitivity of the expectation-maximization (EM) approach to initial parameters, a novel data-driven parameter estimation framework is developed called the Adaptive Expectation-Maximization Attention Algorithm (AEMA). It can autonomously alternate between maximum likelihood estimation and latent variable information interpolation to return the missing information we want while ensuring fast and stable convergence.
3. Actual individual trajectory tracking (ITT) data is used to compare baselines on multiple OD pair datasets, thereby confirming the effectiveness and robustness of the proposed approach, STPGM-AEMA.

The paper is structured into six sections. Section 2 describes the problem of reconstructing individual trajectories with incomplete information. In Section 3, the trajectory inference model is developed, and the methods for parameter estimation are described in Section 4. Section 5 outlines the validation scenarios and compares various methods using real ITT data, followed by an interpretive analysis and a residual analysis of the model results. Finally, Section 6 elucidates the conclusion of the study.

## 2. Problem Description

**Figure 1:**
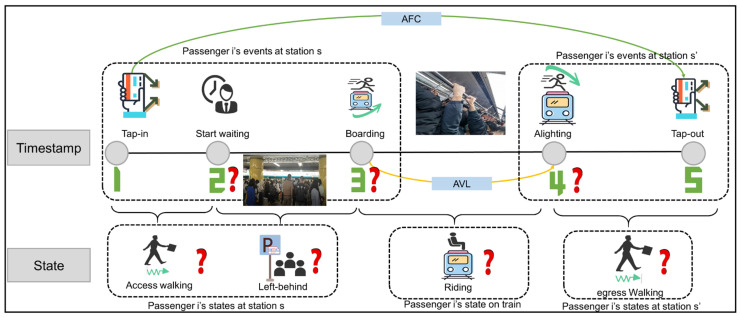
Example of individual travel itinerary.

In the closed URT system, it is assumed that the passenger i enters into the station s at t and leaves from station $s^{\prime }$ at $t^{\prime }$, as exemplified by OD pair on a single line in Figure 1. Only tap-in and tap-out events are recorded with spatiotemporal information from AFC data, and train arrival and departure events are obtained from AVL data. However, due to the low sampling frequency, the sequential events of each passenger, e.g., waiting for boarding event, boarding, and alighting event, and the associated state information, are severely missing in the system. This further results in the inability to obtain accurate system status (e.g., congestion state at platforms or on trains). 

The information lost in a single trajectory is usually obtained through the spatiotemporal interpolation method, but it usually cannot satisfy the comparison of semantic information in URTS. Different from traditional methods, this paper aims to capture the missing spatiotemporal events, status, and semantic information in passenger trajectories through data mining, information interaction design, parameter learning, and probabilistic reasoning without manual investigation. This process is called individual trajectory reconstruction (ITR). It is worth noting that the problem of ITR in this paper is a further extension of individual trajectory prediction at the train level.

Further, a set of journey among OD pairs is defined as $X=\left\{x_{1},\ldots ,x_{I},\ldots ,x_{N}\right\}$, where $x_{I}$ represents the original information that can be obtained, with the index being I∈1,…,N, and the total number of trips being N. Based on this, $D=\left\{D_{1},\ldots ,D_{I},\ldots ,D_{N}\right\}$ is defined as a set of observable information, it comprises known itinerary information $x_{I}$, system observability data $D_{\mathrm{sys}}$, and mined information $D_{\mathrm{mining}}$ encompassing individual trips, train operations, station flows, etc. It can be given as follows:

(1) $$D=\left\{D_{I}\right\}=\left\{X_{I},D_{\mathrm{sys}},D_{\mathrm{mining}}\right\}$$

Next, the individual trajectory $tr_{I}$ is defined as being represented by a sequence of ordered spatiotemporal events E recorded in chronological order and a state vector S. $tr_{I}$ can be stated as follows:

(2) $$tr_{I}=\left\{E,S\right\}=\left\{\left\{E_{h}\right\},\left\{S_{f}\right\}\right\},h\in [1,M],f\in [1,W]$$

where $E_{h}$ denotes a single spatiotemporal event, h is the event index, there are M in total. $S_{f}$ indicates a state set between two adjacent events, including single or multiple status values. The value f is the state set index, there are W in total. Furthermore, a single event $E_{h}$ is represented in the form of a ternary tuple, containing the characteristics of the moment of occurrence, location, and instantaneous behavior, namely:

(3) $$E_{h}=\left(T_{E_{h}}{,L}_{E_{h}}{,B}_{E_{h}}\right)$$

The passenger travel process consists of two main modes of spatial and temporal transitions, i.e., walking within the station or moving with the train. The state chain of an individual trajectory is defined as follows:

(4) $$S_{f}=\left\{S_{station},S_{train}\right\}$$

where, $S_{station}$ and $S_{train}$ represent the set of states of individuals at the origin/interchange/terminal and on the train, respectively. They can be represented by n-tuples. And every state is a scalar.

The overall trajectories Tr is a set composed of ordered spatiotemporal event sequences as $Tr=\left\{tr_{I}\right\}=\left\{tr_{1},tr_{2},\ldots ,tr_{N}\right\}$.

Summarizing, this paper aims to interpolate missing spatiotemporal events in each travel trajectory and complement the semantic state information through probabilistic inference, which can naturally be represented by conditional probabilities $P\left(Tr|X\right)$. To achieve optimal estimation of individual itineraries inference, a probability-based framework is proposed. Within this framework, the core of ITR is reduced to an optimization problem, namely seeking the parameter configuration Θ that maximizes the posterior probability in the parameter space. This optimization problem can be formalized as follows:

(5) $$\arg\max P\left(Tr|X\right)=\arg\max P\left(Tr|D\right)\propto \underset{\Theta }{\arg\max}P(D,?|\Theta )$$

## 3. Methodology

**Figure 2:**
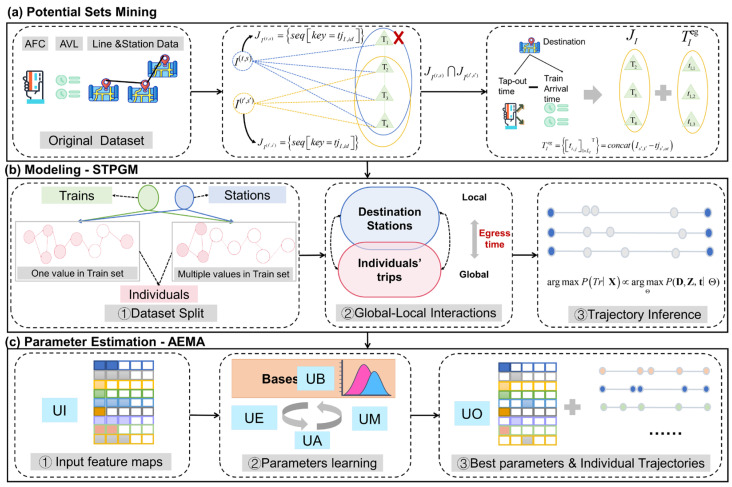
A data-driven spatiotemporal probabilistic graphical model inference framework.

How to make the best use of limited information and infer high-fidelity individual trajectories through appropriate design is the key to methodology. A data-driven spatiotemporal probabilistic graphical model inference framework is proposed in the paper, which consists of three steps: potential set mining, modeling, and parameter estimation. The input data sources of the method are as follows: AFC, AVL, and Lines and Stations data. Where AFC records passengers’ information, including their origin and destination stations and times of entry/exit. AVL data captures train operation information such as the train’s ID, service line, station numbers, and arrival/departure time. Line and Station data provide physical distance and adjacency relations between stations. The outputs are spatiotemporal events and state information involved in individual trajectories. The key steps of the methodology are shown in Figure 2a–c, respectively.

### 3.1. Framework

In brief, the steps are as follows:

1. **Potential Sets Mining.** Considering the sequential nature of passengers’ behaviors in spatiotemporal events, wherein each event is dependent on the preceding specific event, the get-off-leave-now (GOLN) principle is introduced. A feasible train alternative set for a journey as well as an egress time alternative set at the destination station of the individual are obtained, combined with complex spatiotemporal constraints and a combinatorial enumeration algorithm. This strategy can effectively reduce the space of candidate solutions under the premise of guaranteeing accuracy for subsequent computations.
2. **Modeling.** In order to suppress the bias caused by small-sample randomness, global and local latent variables are introduced to model the complex spatiotemporal dependencies of all trips and observed components (stations, trains) in the URT system. The construction of the model consists of three steps: dataset segmentation, global-local interaction representation, and trajectory inference. The main details of the model are presented in Section 3.3.
3. **Parameter Estimation.** To obtain the optimal parameters of the model and infer the most probable trajectories, an adaptive expectation-maximizing attention (AEMA) parameter learning method is proposed, which integrates a base adaptive embedding unit (UB), which provides automated a priori parameters to the likelihood function. Next, the introduction of the key-value attention computation unit (UA), where train labels can be matched to every individual trajectory. Details of the algorithm are given in Section 4.

### 3.2. Potential Sets Mining 

The subsequent section outlines the necessary constraints and computational formulas for resolving both the set of train alternatives and individual travel alternatives. Finally, the combined enumeration method is used to obtain the collection. Appendix A provides relevant notation definitions.

**Constraint** **1.** *The departure time* $tj_{s,dt}$ *of a potential train* tj *at the origin station* s *constraint. The departure time* $tj_{s,dt}$ *must be such that between the time period tap-in time* t *and tap-out time* $t^{\prime }$ *in itinerary* I.

(6) $$I_{s,t}<tj_{s,dt}<I_{s^{\prime },t^{\prime }},tj_{s,at}\neq tj_{s,dt}\;and\;tj_{s,q}\neq tj_{\mathrm{es},q}$$

The process generates a set of potential candidates for the train at the origin s called $J_{I^{\left(t,s\right)}}$:

(7) $$J_{I^{\left(t,s\right)}}=\left\{seq\left[key=tj_{id}\right]\right\}$$

**Constraint** **2.** *The departure time* $tj_{s^{\prime },dt}$ *of the potential train* tj *at the destination station* $s^{\prime }$ *constraint. The departure time* $tj_{s^{\prime },dt}$ *must be such that between the time period tap-in time* t *and tap-out time* $t^{\prime }$ *in itinerary* I.

(8) $$I_{s,t}<tj_{s^{\prime },dt}<I_{s^{\prime },t^{\prime }},tj_{s^{\prime },at}\neq tj_{s^{\prime },dt}\;and\;tj_{s^{\prime },q}\neq tj_{\mathrm{fs},q}$$

A set of potential candidates for the train at destination $s^{\prime }$ can be generated called $J_{I^{\left(t^{\prime },s^{\prime }\right)}}$:

(9) $$J_{I^{\left(t^{\prime },s^{\prime }\right)}}=\left\{seq\left[key=tj_{id}\right]\right\}$$

The set of feasible train choices in the itinerary I can be obtained by taking the intersection, denoted as $J_{I}$:

(10) $$J_{I}=J_{I^{\left(t,s\right)}}\cap J_{I^{\left(t^{\prime },s^{\prime }\right)}}=\left\{j=seq{\left[key=tj_{I,id}\right]}_{1\times L_{I}}\right\},j\in [1,\cdots ,L_{I}]$$

Based on this premise, constructing the egress time sequence set in the itinerary I as $T_{I}^{eg}$. Each egress time value $t_{i,j}$ is calculated as the time difference between the tap-out time and the arrival time $j_{s^{\prime },at}$ of the corresponding train of $J_{I}$.

(11) $$T_{I}^{eg}=\left\{{{\left[t_{i,j}\right]}_{1\times L_{I}}}^{T}\right\}=concat\left(I_{s^{\prime },t^{\prime }}-tj_{s^{\prime },at}\right)$$

### 3.3. Modeling

**Figure 3:**
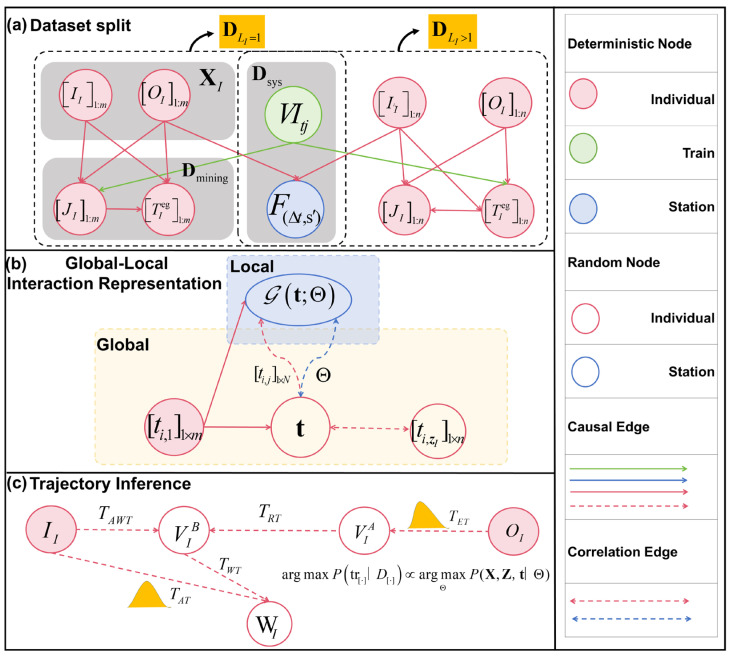
Trajectory inference framework based on STPGM.

The Bayes theorem principle and the backward inference method are introduced to establish a mechanism for global and local interactive representation. After obtaining the optimal parameters, probabilistic reasoning about individual trajectories is realized. Figure 3 illustrates the trajectory inference framework based on STPGM, where color coding is employed to denote different categories of nodes and edges (refer to the legend for details). Events are represented as nodes, while edges describe potential spatial transition dependencies between state time intervals and events. Shaded nodes correspond to deterministic variables, whereas hollow circles indicate unobservable random variables. Solid and dashed lines distinguish deterministic relationships from uncertain ones, with unidirectional arrows representing causal relationships and bidirectional arrows indicating correlations.

As Equation (3), the set of nodes state as follows:

(12) $$\left\{E_{h}\right\}=\left\{I_{I},W_{I},V_{I}^{B},V_{I}^{A},O_{I}\right\}$$

where, the events of tap-in $I_{I}$, waiting for boarding $W_{I}$, boarding $V_{I}^{B}$, alighting $V_{I}^{A}$, and tap-out $O_{I}$ are represented in sequential order. 

As in Equations (4) and (12), for OD pairs on a single line that do not require transfers, the state chain of an individual trajectory is defined as follows:

(13) $$\left\{S_{f}\right\}=\left\{S_{s},S_{j},S_{s^{\prime }}\right\}=\left\{\left(T_{AWT},T_{WT},T_{AT}\right),\left(T_{RT}\right),\left(T_{ET}\right)\right\}$$

where, $S_{s},S_{j},S_{s^{\prime }}$ represents the state of an individual at different spatial locations of the origin station s, train j, and destination station $s^{\prime }$, respectively. The value of the total time at the origin station $T_{AWT}$ is calculated by summing the access time $T_{AT}$ and the waiting time $T_{WT}$. $T_{RT}$ denotes the running time on the train and $T_{ET}$ indicates the egress time at the destination station. An individual trajectory $tr_{I}$ can be represented as follows:

$$tr_{I}=\left\{E=\left\{\begin{array}{l} I_{I}=\left(08:00:23,TTYB,\mathrm{Tap}-in\right)\\ W_{I}=\left(08:01:28,TTYB,Start\;waiting\right)\\ V_{I}^{B}=\left(08:04:32,TTYB,Boarding\;Train\;j\right)\\ V_{I}^{A}=\left(08:28:20,DD,Alighting\right)\\ O_{I}=\left(08:29:03,DD,\mathrm{Tap}-out\right) \end{array}\right\},S=\left\{\begin{array}{l} S_{s}=\left(249s,65s,184s\right)\\ S_{j}=\left(1428s\right)\\ S_{s^{\prime }}=\left(43s\right) \end{array}\right\}\right\}.$$

The inference tasks of this paper encompass the identification of waiting events at the platform, as well as the boarding and alighting events of passengers, along with a chain of unknown states. To establish the model, two strategies are employed:

1. The data is divided into deterministic dataset $D_{L_{I}=1}$ and stochastic dataset $D_{L_{I}>1}$ in order to generate prior samples.
2. A global-local interaction module is devised to transform the problem from maximizing the probability of individual trajectories to posterior parameter estimation based on the basis function. Building upon this foundation, boarding and alighting events are inferred by estimating egress time $T_{ET}$, then determining access time $T_{AT}$ and waiting for the event through MCMC simulation, thereby achieving comprehensive inference of unknown events and latent states in trajectories. The modeling process consists of three steps which are described in detail below.

#### 3.3.1. Dataset Split

In this paper, the dataset is split into a deterministic dataset $D_{L_{I}=1}$ and a stochastic dataset $D_{L_{I}>1}$ with multiple alternatives, based on whether the number of options in the train candidate set is greater than one. Consequently, Equation (1) can be modified accordingly:

(14) $$D=\left\{\left(D_{L_{I}=1},I=1,\ldots ,m),(D_{L_{I}>1},I=1,\ldots ,n\right)\right\}$$

Wherein, the numbers of samples in the deterministic dataset and the stochastic dataset are respectively denoted as m and n, with m+n=N. This approach benefits by providing prior data for the training of model parameters from the deterministic dataset $D_{L_{I}=1}$, thereby replacing manual surveys and reducing the introduction of system noise.

Observable information is redefined based on node information, as shown in the dashed box on the left side of Figure 3a, taking the observable dataset as an example:

(15) $$D_{L_{I}=1}={\left\{x_{I},D_{\mathrm{sys}},D_{\mathrm{mining}}\right\}}_{L_{I}=1}=\left\{{\left[I_{I},O_{I}\right]}_{1:m},\left[\left(VI_{tj}\right),\left(F_{\left(\Delta t,s^{\prime }\right)}\right)\right],{\left[J_{I},T_{I}^{\mathrm{eg}}\right]}_{1:m}\right\}$$

where, an individual’s journey $x_{I}$ observations encompass tap-in event $I_{I}$ and tap-out $O_{I}$ event, while system observations $D_{\mathrm{sys}}$ include train operation events $VI_{tj}$ and outbound passenger flow within a specific time interval $F_{\left(\Delta t,s^{\prime }\right)}$. The mined information set $D_{\mathrm{mining}}$ comprises a feasible train choices set $J_{I}$ and potential egress time set $T_{I}^{\mathrm{eg}}$, with their sample sizes remaining consistent. It is important to note that these observable pieces of information are either localized or aggregated. Similarly, this definition $D_{L_{I}>1}$ follows a similar logical framework.

#### 3.3.2. Global-Local Interactive Representation

In the study of passenger journeys between OD pairs under incomplete information, the spatiotemporal dependency is manifested in the dynamics of individual travel events and state information as they evolve over time and space, exerting a significant influence on the local elements of the system. This paper introduces two latent variables to facilitate parameter estimation based on local elements, as elaborated below.

**Global variable: latent variables** ${𝓩}_{I}$ **and** t. The index position corresponding to the individual egress time $t_{i,j}$ is set as a discrete random hidden variable ${𝓩}_{I}$, following a multinomial distribution. The probability mass function can be expressed as follows:

(16) $$P\left({𝓩}_{I}=j\right)=p_{ij},j=1,2,\cdots L_{I}$$

where, $p_{ij}$ represents the probability of selecting the jth index in $T_{I}^{\mathrm{eg}}$, and satisfies $\sum_{j=1}^{L_{I}}p_{ij}=1$, represents the probability distribution in the ordered sequence $j=1,2,\cdots L_{I}$. The complete hidden variable is denoted as $Z=\left\{{𝓩}_{I}\right\}$.

Moreover, in order to effectively characterize the parameter variations throughout the iterative process and disentangle the interdependencies between global and local elements, we propose a set of aggregate vectors referred to t, which are composed of egress time $t_{i,j}$ for all individuals. Consequently, we obtain the following:

(17) $$t={\left[t_{i,j}\right]}_{1\times N}=c\left({\left[t_{i,1}\right]}_{1\times m},{\left[t_{i,z_{I}}\right]}_{1\times n}\right),t_{i,1}\in D_{L_{I}=1},t_{i,z_{I}}\in D_{L_{I}>1}$$

where, $t_{i,1}$ represents the unique egress time value from dataset $D_{L_{I}=1}$, with the dimension being 1×m; $t_{i,{𝓩}_{I}}$ states the $j\mathrm{th}(z_{I})$ egress time component from the set $T_{I}^{\mathrm{eg}}$ of $D_{L_{I}>1}$, with the dimension being 1×n; and c denotes the vector concatenation operation.

**Local variable: basis function** $G\left(\cdot \right)$**.** In this paper, the distribution of egress time $G\left(\cdot \right)$ is designed as a local variable. It is represented by a continuous probability distribution form that is integrable $\int_{0}^{\infty }x\cdot f(x)dx$ and $\int_{0}^{\infty }{\left(x-\mu \right)}^{2}\cdot f(x)dx$ absolutely convergent, meaning it possesses finite mean and variance as a basis function $G\left(t;\Theta \right)$. The general form of representation is provided as follows:

(18) $$G\left(t;\Theta \right)=G\left(t;\theta ,\mu ,\sigma^{2}\right)$$

where, Θ represents parameters related to the time scale △t and exit station $s^{\prime }$, functioning as spatiotemporally adaptive parameters. θ denotes the intrinsic parameters of the function $G\left(\cdot \right)$ itself, μ determines the central position of the distribution, $\sigma^{2}$ describes the dispersion of data points around the mean, and t signifies the input value.

**Interactive representation mechanism.** Figure 3b shows the global-local interactive representation mechanism by basis function $G\left(\cdot \right)$, latent variables ${𝓩}_{I}$, and t. Among them, vector t plays a key role. As a transmission channel, it not only aggregates the egress time information of all individuals but also provides the required input for updating the parameters of the basis function $G\left(\cdot \right)$.

Specifically, in the process of transferring information from global parameters Θ to local parameters ${𝓩}_{I}$, t collects the candidate egress time $t_{i,z_{I}}$ generated by each individual in each iteration, passes these data to the function $G\left(\cdot \right)$, and estimates by MLE to fit the parameters Θ. This step ensures that local parameter updates reflect the latest data in the context of the system.

In turn, the results of parameter optimization are used to construct the query vector and the global variable candidate solution $t_{i,z_{I}}$ is used as the key component to construct the key-value pair for the next step of similarity comparison (to be described in detail in Section 4). Through this operation, we can re-evaluate and update everyone’s $t_{i,z_{I}}$, thereby optimizing the performance of the entire system in each iteration.

#### 3.3.3. Trajectory Inference

**1. Calculate the maximum probability of individual trajectories.** Under the principle GOLN, the problem of calculating the maximum probability of all journeys is equivalent to estimating the best parameters $\Theta^{*}$ of the basis function $G\left(\cdot \right)$ by maximizing the probability of t under the influence of the latent variable Z, making the observed data most likely to occur. Thus:

(19) $$\arg\max P\left(Tr|X\right)\propto \underset{\Theta }{\arg\max}P(D,Z,t|\Theta )$$

It is worth noting that the basis vector t serves as a conduit, facilitating the process of global-local interaction by aggregating individual travel time information and channeling it to the basis function $G\left(\cdot \right)$ representing local characteristics. Subsequently, parameter updates occur during each iteration to ensure stability in parameter estimation, which is elaborated upon in Section 4. 

**2. Calculate unknown events and state variables.** Figure 3c illustrates the trajectory inference process, indicating that the egress time of the passenger is $T_{ET}=t_{i,z_{I}}$. Subsequently, the individual’s train ID is determined as follows:

(20) $$tj_{I,id}=idx[i,j]$$

where, idx[·] represents a mapping function used to locate an element based on its index number. Further, the spatiotemporal characteristics of the boarding event $V_{I}^{B}$ and alighting event $V_{I}^{A}$ are established, and the running time duration and access time are computed by $T_{RT}=T_{V_{I}^{A}}-T_{V_{I}^{B}}$ and $T_{AWT}=T_{V_{I}^{B}}-T_{I_{I}}$, individually.

The next step is to use the formula quoted by Zhu et al. [20] to calculate the waiting time $T_{WT}$, which is a typical formula. And the access time of every passenger can be calculated by $T_{AT}=T_{AWT}-T_{WT}$. However, it was found that about 10% of the results of $T_{AT}$ are negative by calculating $T_{WT}$ and $T_{AT}$, using 26,000 samples from one line of the Beijing Rail Transit System (BRTS) during the peak period. This reveals that this method may not be suitable for BRTS. Building upon the decomposition method outlined in Equation (14), this study defines the waiting duration of the sample set $x_{I}\in X_{L_{I}=1}$ as follows:

(21) $$T_{WT}\left(x_{I}\in X_{L_{I}=1}\right)=\frac{E(H_{j\to j+1})}{2}+\frac{\mathrm{Var}(H_{j\to j+1})}{2E(H_{j\to j+1})}$$

where, $H_{j\to j+1}$ states the departure interval between jth and j+1th train.

The access time of the sample set $x_{I}\in X_{L_{I}=1}$ is calculated by means of a piecewise function as follows:

(22) $$T_{AT}\left(x_{I}\in X_{L_{I}=1}\right)=\left\{\begin{array}{c} T_{AWT}-T_{WT},\;if\;T_{AWT}>T_{WT}\\ T_{AWT},\;\;\;\;\;\;\;else\;\; \end{array}\right.$$

On this basis, the waiting duration of the sample set $x_{I}\in X_{L_{I}>1}$ calculated by the following:

(23) $$T_{WT}\left(x_{I}\in X_{L_{I}>1}\right)=T_{AWT}-T_{AT}$$

## 4. Parameter Estimation

The second challenge addressed in this paper is how to design a likelihood function that maximizes the reconstruction of high-fidelity trajectories for all individuals Tr, considering dependence between t and Θ. To tackle this, we draw inspiration from the GMM for mixed distributions [35] and the EMA for semantic segmentation [44]. The Adaptive Expectation-Maximization Attention (AEMA) algorithm is proposed, which incorporates the EM algorithm and attention mechanisms. 

The idea behind the proposed algorithm is derived from how to establish a data flow mechanism between global variables representing all individuals and local features representing the system, which is crucial for fully data-driven algorithms.

**Figure 4:**
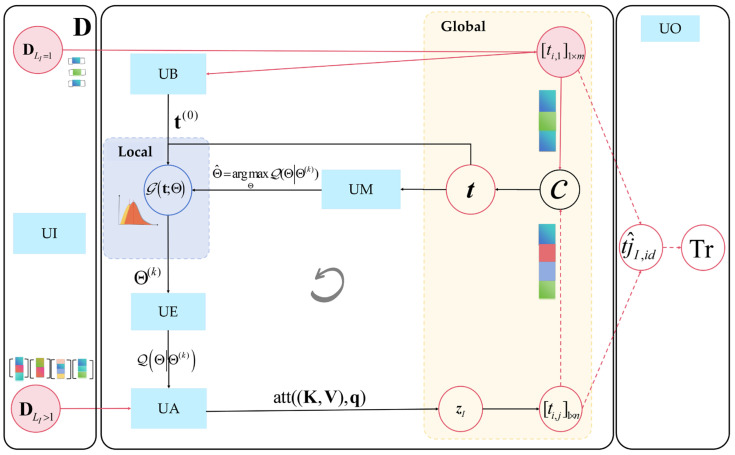
Parametric learning process based on the AEMA algorithm.

The AEMA algorithm consists of an input and output unit and four main operation units (as shown in Figure 4), namely: Input Unit (UI), Bases Adaptability Embedding Unit (UB), Expectation-Step Unit (UE), Key-Value Attention Calculation Unit (UA), Maximization-Step Unit (UM), and Output Unit (UO). In brief, the UI is the first step of AEMA, aimed at providing the UB with observable dataset inputs. The UB is responsible for dynamically obtaining initial base vectors providing an initial parameter for fitting the distribution. UE, the E-step in the EM algorithm, defines the objective function under prior parameters. UA provides methods for computing the posterior distribution of hidden variables. UM, the M-step in the EM algorithm, aims to maximize until convergence criteria are met. The UO outputs the reconstructed trajectories. Each step is explained below.

### 4.1. Input Unit (UI)

UI is responsible for inputting the observable data set and defining the parameters of the core steps. It is important to note that $\left\{X,\left[t_{i,j}\right],\left[\left[z_{i,j}\right]\right]\right\}$ should be considered as complete observation data, with the parameters to be estimated as $\Theta =\{\theta ,\mu ,\sigma^{2},\left[\left[p_{ij}\right]\right]\}$, and the number of parameters as $3+N*L_{I}$. Let the likelihood function be $L\left(\Theta \right)=P(D,Z,t|\Theta )$, with the conditional distribution of the latent variables Z and t being $P(Z,t|D,\Theta^{\left(k\right)})$. Where $\Theta^{\left(k\right)}$ represents the parameter estimated in the kth iteration. The parameters in the k+1th round, $\Theta^{*}$ are thus the target parameter values to be maximized.

### 4.2. Bases Adaptability Embedding Unit (UB)

Prior information is typically obtained through surveys involving small labeled datasets, followed by fitting the parameters $\Theta^{\left(0\right)}$ through maximum likelihood estimation (MLE) [20]. However, the calculation of this mean value μ cannot be adaptively chosen, and different scenarios require different survey data. UB is responsible for acquiring prior information and initializing parameters. It dynamically obtains samples from the dataset $D_{L_{I}=1}$ based on station $s^{\prime }$ and time interval △t as inputs for prior knowledge, supporting the automated calculation of prior parameters $\Theta^{\left(0\right)}$ for using the MLE method, replacing the practice in traditional EM algorithms of randomly initializing model parameters. Let:

(24) $$\Theta^{\left(0\right)}=\underset{\Theta }{\arg\max}\prod_{i=1}^{m}G\left(\Theta \left|{\left[t_{i,1}\right]}_{1\times m}\right.\right),t_{i,1}\in D_{L_{I}=1}$$

In summary, the UB exhibits the capability to automatically capture spatiotemporal information thereby enhancing the model’s robustness and accuracy in handling intricate spatiotemporal correlations and dynamic patterns. This effectively addresses the issue of sensitivity in parameter initialization. 

### 4.3. Expectation-Step Unit (UE)

The marginal likelihood function of a sample is denoted as $p(D_{I},{𝓩}_{I},t_{i,j}|\Theta )$, while the conditional distribution probability of the latent variables is represented by $p({𝓩}_{I},t_{i,j}|D_{I},\Theta^{\left(k\right)})$. By applying Jensen’s inequality, the log-likelihood function is:

(25) $$\begin{array}{ll} \ln L\left(\Theta \right) & =\ln\sum_{Z}p(D_{I},Z,t_{i,j}|\Theta )=\sum_{N}\sum_{{𝓩}_{I}}\ln p(D_{I},{𝓩}_{i,j},t_{i,j}|\Theta )\\ & =\sum_{N}\sum_{{𝓩}_{I}}\ln\frac{p(D_{I},{𝓩}_{i,j},t_{i,j}|\Theta^{\left(k\right)})\cdot p({𝓩}_{i,j},t_{i,j}|D_{I},\Theta^{\left(k\right)})}{p({𝓩}_{i,j},t_{i,j}|D_{I},\Theta^{\left(k\right)})}\\ & \geq \sum_{N}\sum_{{𝓩}_{I}}p({𝓩}_{i,j},t_{i,j}|D_{I},\Theta^{\left(k\right)})\ln p(D_{I},{𝓩}_{i,j},t_{i,j}|\Theta )/p(D_{I},{𝓩}_{i,j},t_{i,j}|\Theta^{\left(k\right)})\\ & =\sum_{N}\sum_{{𝓩}_{I}}p({𝓩}_{i,j},t_{i,j}|D_{I},\Theta^{\left(k\right)})\ln p(D_{I},{𝓩}_{i,j},t_{i,j}|\Theta )-\sum_{N}\sum_{{𝓩}_{I}}p({𝓩}_{i,j},t_{i,j}|D_{I},\Theta^{\left(k\right)})\ln p(D_{I},{𝓩}_{i,j},t_{i,j}|\Theta^{\left(k\right)}) \end{array}$$

where $\sum_{N}\sum_{{𝓩}_{I}}p({𝓩}_{i,j},t_{i,j}|D_{I},\Theta^{\left(k\right)})\ln p(D_{I},{𝓩}_{i,j},t_{i,j}|\Theta^{\left(k\right)})$ is a constant, then the objective function is $𝓠\left(\Theta \left|\Theta^{\left(k\right)}\right.\right)$, which is given by the following:

(26) $$\begin{array}{l} 𝓠\left(\Theta \left|\Theta^{\left(k\right)}\right.\right)=\sum_{N}\sum_{{𝓩}_{I}}p({𝓩}_{i,j},t_{i,j}|D_{I},\Theta^{\left(k\right)})\ln p(D_{I},{𝓩}_{i,j},t_{i,j}|\Theta )\\ =E_{Z}[\ln P(D,Z|\Theta )|D,\Theta^{\left(k\right)}] \end{array}$$

It is evident that we need to calculate the conditional distribution probability $p({𝓩}_{I},t_{i,j}|D_{I},\Theta^{\left(k\right)})$ for each individual and use it as the maximization target function $𝓠\left(\Theta \left|\Theta^{\left(k\right)}\right.\right)$ in the UM. However, due to the nonlinearity of high-order terms in the objective function, conventional optimization methods may be sensitive to initial parameters and prone to local optima. To more effectively capture the relationship between an individual’s egress time sequence set $T_{I}^{\mathrm{eg}}$ and the station’s local prior information while reducing the communication cost, this paper draws on a key-value attention mechanism. This involves constructing a query vector representing local parameters and determining attention weights for key-value pairs $\left(k_{I},v_{I}\right)$ associated with each individual during the UA step.

### 4.4. Key-Value Attention Calculation Unit (UA) 

**Figure 5:**
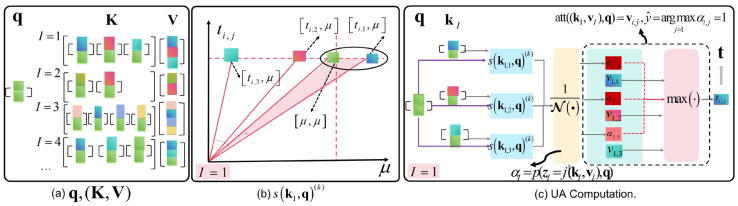
Calculation process of key-value attention unit (UA).

In this step, the distribution of the latent variables Z and t are calculated. Figure 5 shows the entire calculation process of UA. The specific steps are as follows:

First, the query vector (as shown in Figure 5a). It is constructed by leveraging the broadcasting method commonly used in deep learning to transform scalar values into a vector representation. Specifically, we utilize the parameter u to create a vectorized form denoted as $q_{T_{\left(△t,s^{\prime }\right)}^{eg}}^{\left(k\right)}$, which stands for q. Let:

(27) $$q_{T_{\left(△t,s^{\prime }\right)}^{eg}}^{\left(k\right)}={\left[\mu^{\left(k\right)},\mu^{\left(k\right)}\right]}^{T},\forall \mu \in \Theta$$

In fact, this definition expands the information aggregated at the current station. Furthermore, the initial value $q^{\left(0\right)}$ comes from the sample set $D_{L_{I}=1}$.

Second, key-value pairs (as shown in Figure 5a). Let $k_{I}={\left[{\left[t_{i,1},\mu^{\left(k\right)}\right]}^{T},\cdots ,{\left[t_{i,j},\mu^{\left(k\right)}\right]}^{T}\right]}_{1\times L_{I}}$ used to simulate the values that each individual with multiple potential trajectories can take in each round, and let $v_{I}={\left[\begin{array}{ccc} t_{i,1} & \cdots & t_{i,j} \end{array}\right]}_{1\times L_{I}}^{T}$ represent the egress time corresponding to the index position of the latent variable. So there are:

(28) $${\left(K,V\right)}^{\left(k\right)}={\left\{\left(k_{I},v_{I}\right)\right\}}^{\left(k\right)},I\in [1,\ldots ,n]$$

Third, the scoring function is defined (as depicted in Figure 5b). The role of this function is to compute the correlation between each input vector $k_{I}$ and the query vector q. In the standard Scaled Dot Product Model, the dot product operation tends to be more sensitive to a larger value. This study focuses on computing proximity values between vectors $k_{I}$ and q for assigning higher weights accordingly. Hence, cosine similarity based on vector angle principles is chosen as the definition for the scoring function. This approach not only considers individual value relationships with groups but also accounts for self-relationship. Let:

(29) $$s{\left(k_{I},q\right)}^{\left(k\right)}=\frac{k_{I}\cdot q}{\left\|k_{I}\right\|\cdot \left\|q\right\|}$$

where, the inner product of vectors, denoted by $k_{I}\cdot q$, $\left\|\cdot \right\|$ represents the norm of a vector. A smaller angle indicates higher similarity.

Moreover, calculate the attention function (as shown in Figure 5c). The attention distribution $\alpha_{I}$ represents the degree of attention the jth component of $k_{I}$, given the query vector q. Specifically, when dealing with class-imbalanced data, traditional attention functions such as $Softmax\left(\cdot \right)$ often fail to provide sufficient learning opportunities for minority classes because they may be suppressed by dominant classes during computation. To overcome this, we use a normalization function $N\left(\cdot \right)$ to replace the traditional activation function, which can enhance the model’s focus on minority class features and learning efficiency. The calculation of $\alpha_{I}$ amounts to computing the posterior probability distribution of ${𝓩}_{I}$, which is as follows:

(30) $$\alpha_{I}=[\alpha_{i,j}]=p({𝓩}_{I}=j|(k_{I},v_{I}),q)=\frac{s\left(k_{I},q\right)}{N\left(\cdot \right)}=s\left(k_{I},q\right)/\sum_{1}^{n}s\left(k_{I},q\right)$$

The attention mechanism offers two available forms, hard attention is chosen in this paper. Subsequently, the attention function is defined as:

(31) $$\mathrm{att}((k_{I},v_{I}),q)=v_{i,\hat{y}}=[t_{i,\hat{y}}],\hat{y}=\underset{j=1}{\arg\max}\alpha_{i,j}$$

where $\hat{y}$ is the subscript of the input vector $v_{i,j}$ with the greatest probability, name is $\underset{j=1}{\arg\max}\alpha_{i,j}$.

Finally, the basis variable t is calculated, let:

(32) $$t={\left[t_{i,j}\right]}_{1\times N}=c\left({\left[t_{i,1}\right]}_{1\times m},{\left[t_{i,\hat{y}}\right]}_{1\times n}\right),t_{i,1}\in D_{L_{I}=1}$$

### 4.5. Maximization-Step Unit (UM)

The main objective in this step is to solve the optimization problem by utilizing the complete observed variables and obtaining maximum likelihood estimates of the parameters, which is given by Equation (26). Let:

(33) $$\Theta^{*}=\underset{\Theta }{\arg\max}\;𝓠\left(\Theta \left|\Theta^{\left(k\right)}\right.\right)$$

Finally, by alternating iterations among the UE, UA, and UM until convergence is achieved. In this process, three termination conditions are set. (1) The local variable Θ is controlled by the Tolerance parameter $\epsilon_{1}$ (set to 1 × 10^−3^). (2) The global variables convergence tolerance is managed by the tolerance of the objective function $\epsilon_{2}$ (set to 1 × 10^−1^). (3) The overall convergence speed is controlled by setting a maximum number of iterations K, which is set to 50 times. If the algorithm satisfies the tolerance conditions before reaching the maximum number of iterations, it will stop prematurely. That is as follows:

(34) $$\left\{\begin{array}{l} \left\|\Theta^{\left(k+1\right)}-\Theta^{\left(k\right)}\right\|<\epsilon_{1}\;\mathrm{or}\;\\ Q\left(\Theta^{\left(k+1\right)},\Theta^{\left(k\right)}\right)-Q\left(\Theta^{\left(k\right)},\Theta^{\left(k\right)}\right)<\epsilon_{2}\;\mathrm{or}\\ k=K \end{array}\right.$$

### 4.6. Output Unit (UO)

The train ID serves as a primary criterion for assessing the accuracy of the spatial position of trajectories in ITR. According to Equation (18), it is known that $t{\hat{j}}_{I,id}=idx[i,\hat{y}]$. Further, the samples for $x_{I}\in X_{L_{I}>1}$ are simulated to generate values $T_{AT}$ using the NUTS algorithm for MCMC sampling with the pyMC3 library. Based on this, by applying the formulas from Section 3.3.3, we can provide detailed information on the attributes and state features of unknown events involved for all passengers.

## 5. Experiments

In traditional methods, local features (like egress time distribution) are simulated to verify the accuracy of parameters or the usability of methods [12,42], but they are rarely considered from the perspective of actual individual trajectories. Verifying the method from a bottom-level rather than an aggregate perspective is also one of the contributions of this article.

### 5.1. Dataset Description

#### 5.1.1. Design of Individual Trajectory Tracking Simulation Experiment

During peak hours, a large number of passengers entering the origin station or transferring at the transfer station will flow into the platform; meanwhile, many passengers will flow out at other destination stations. This may cause local congestion and increase the complexity of the spatiotemporal modeling of travel trajectories. Therefore, in the design stage of the individual trajectory tracking simulation experiment, this study pays special attention to capturing the tidal characteristics of the passenger flow [35]. Take the CY station in Line 6 as an example. The station is located in a suburban area with more residential areas in the neighborhood, and the main service targets are commuters. During the morning peak period, most passengers entering the station from 7:00 to 09:00 are heading towards downtown Beijing, and the passenger inflow during this period accounts for about 50% of the total daily passenger inflow. In contrast, the number of passengers entering the station from 17:00 to 20:00 in the evening is significantly lower, accounting for only about 15% of the total. 

In addition, in order to better validate the effectiveness and applicability of the STPGM-AEMA method proposed in this article, several factors are considered in depth in this study. First, this study covers OD pairs with different distances and station types to ensure the comprehensiveness and representativeness of the experimental setup. Moreover, the investigation team is from Beijing Metro Network Management Co. Ltd., Beijing, China, the official regulator of the BRTS. Considering the human and material conditions coordinated by this agency, we carefully selected four typical OD pairs for experimental validation. See Section 5.1.2 for details.

**Figure 6:**
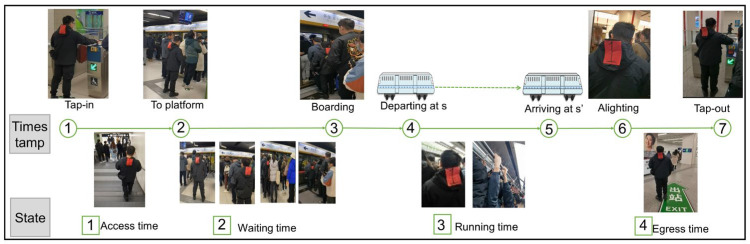
Actual trajectory to obtain experimental records.

Next, in the actual simulation phase, the investigation team simulates the travel process of actual passengers on specific OD pairs and obtained data on each timestamp on the travel chain (as shown in Figure 6), including tap-in time, time of arrival at the platform, time of train departure at the origin station, boarding time, time of train arrival at the destination station, alighting time, and tap-out time. Simultaneously, the integration of AFC and AVL data was employed to generate travel chain status information encompassing behavioral semantics such as access time to the platform, waiting time, running time, and egress time at the destination station. The timestamp and state data tables are provided in Appendix B and Appendix C, respectively, serving as a genuine and dependable foundation for evaluating the efficacy of the proposed methodology.

#### 5.1.2. Data Introduction

**Figure 7:**
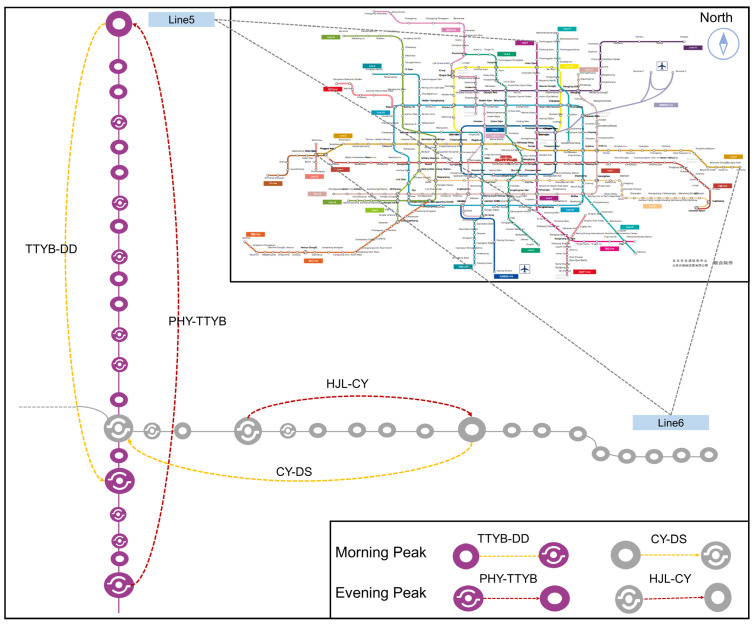
Typical OD pairs.

As an illustration in Figure 7, typical OD pairs from Line 5 and Line 6 of the BRTS during peak periods were selected. The complete dataset comprises four data groups (D1~D4) collected in March 2023, including two morning peak period OD pairs (TTYB-DD, CY-DS) and two evening peak period OD pairs (PHY-TTYB, HJL-CY). Table 1 presents information related to the dataset that encompasses three parts: basic information, validation data information, and training data information. Basic information includes route details, such as the number of stations and distance covered. Validation data eliminates 10 trajectories that do not meet requirements, with a total sample size of ITT of 78. It is noteworthy that AFC and AVL data must correspond one-to-one in terms of dates and periods, while ITT data is included in all AFC data for training purposes. Training data involves multiple days, with a total of 3851 AFC samples and 1069 AVL samples.

**Table 1:** Dataset Description.

Description	Dataset	D1	D2	D3	D4
I. Base Information	OD pair	TTYB-DD	CY-DS	PHY-TTYB	HJL-CY
	Line	5	6	5	6
	Station Numbers	17	10	21	7
	Distance(m)	19,700	15,771	24,480	11,859
II. Validation Data	Time Duration	07:00–09:00 a.m.		17:00–19:00	
	Day	2023.03.21	2023.03.22	2023.03.21	2023.03.22
	ITT Numbers	21	19	20	18
III. Training Data	Days	6	6	4	4
	AFC Samples	1359	699	206	1587
	Train Numbers	340	309	225	195

The parameter learning process involves training utilizing AEMA, followed by performing probabilistic inference and subsequently comparing the inference results with the timestamp and status information of the verification data. The L-BFGS-B optimization algorithm is predominantly employed for parameter learning among various comparison algorithms. All algorithms are compiled and executed on a computer equipped with an Intel(R) Core(TM) i9-10920X CPU processor and 48 GB of memory.

### 5.2. Baselines

This paper compares the proposed method, STPGM-AEMA, with traditional rule-based approaches and Bayesian methods at the train-level scale. The methods are outlined as follows:

1. **LTRM (Last Train Rule-based Model):** Spatiotemporal Segmentation of Metro Trips algorithm searching for “BORDER-WALKERS” using the nearest timestamp principle, proposed by Zhang et al. [15], wherein the train’s departure time closest to the passenger’s tap-out time at the destination station was utilized to determine the train they boarded. Luo et al. [45] also employed this rule to infer passenger trajectories. Furthermore, both studies assumed “speed invariance” as a behavioral postulate.
2. **PTAM-MLE (Passenger-to-Train Assignment Model with MLE)**: Zhu et al. [20] proposed a probabilistic approach, named PTAM, which requires AFC/AVL data and the station’s walking speed distribution as inputs. To ensure consistency in measuring speed, this paper replaces it with their later proposed LBPMF [42], where the input is the egress/access time distribution and the likelihood function is expressed accordingly.
3. **MPTAM-EM (Modified Passenger-to-Train Assignment Model with EM)**: A modified model MPTAM was constructed by Xiong et al. [12], and the EM algorithm was proposed for estimating the parameters of the egress time distribution and the boarding probability distribution function, and the likelihood function was formulated by them.
4. **STPGM-EMA (without UB)**: The proposed STPGM-AEMA algorithm forms the basis of this method, which entails the removal of the UB module.
5. **STPGM-AEM (without UA)**: Similarly, the proposed STPGM-AEMA algorithm forms the basis of this method, which entails the removal of the UA module.

### 5.3. Evaluation Metrics

The present study employs two categories of metrics to assess its accuracy and robustness. The details are given below.

#### 5.3.1. Accuracy Evaluation Metrics

Considering the precision of evaluating the multiclassification problem, a confusion matrix is introduced, and six evaluation metrics are chosen: macro-precision, macro-recall, macro-F1 score, micro-precision, micro-recall, and micro-F1 score, and calculated based on true positives (TP), false positives (FP), and false negatives (FN) across all categories. These metrics serve as indicators for model performance improvement; higher values indicate better results. The formula is as follows:

(35) $$P_{Macro}=\sum_{c=1}^{N}P_{c}/N$$

(36) $$R_{Macro}=\sum_{c=1}^{N}R_{c}/N$$

(37) $$F1_{Macro}=\sum_{c=1}^{N}F1_{c}/N$$

(38) $$P_{Micro}=\sum_{c=1}^{N}TP_{c}/\left(\sum_{c=1}^{N}TP_{c}+\sum_{c=1}^{N}FP_{c}\right)$$

(39) $$R_{Micro}=\sum_{c=1}^{k}TP_{c}/\left(\sum_{c=1}^{N}TP_{c}+\sum_{c=1}^{N}FN_{c}\right)$$

(40) $$F1_{Micro}=2\cdot \frac{\;P_{Micro}\;\cdot R_{Micro}}{\;P_{Micro}+R_{Micro}\;}$$

where, c is the index of categories.

#### 5.3.2. Consistency Evaluation Metric 

Considering the impact of random classification by the model, this paper introduces Cohen’s Kappa coefficient (K) to calculate the overall consistency and random agreement between observed and predicted values. The *K* measures the model’s resistance to interference in the presence of a class imbalance, serving as a statistical measure to evaluate credibility. The formula is as follows:

(41) $$K=\frac{P_{o}-P_{e}}{1-P_{e}}$$

where, the variable $P_{o}$ represents the observed accuracy, i.e., the proportion of correctly classified instances. $P_{e}$ denotes the expected accuracy, which refers to the proportion of instances correctly classified by chance. The *K* value ranges between [−1, 1], with higher values indicating a model’s more genuine resistance to randomness. A *K* value closer to 1 signifies a model’s perfect agreement with reality; *K* = 0 indicates the model’s performance is equivalent to random classification; and *K* < 0 suggests the model’s performance is even worse than random classification.

### 5.4. Result

#### 5.4.1. Accuracy Evaluation Results

**Figure 8:**
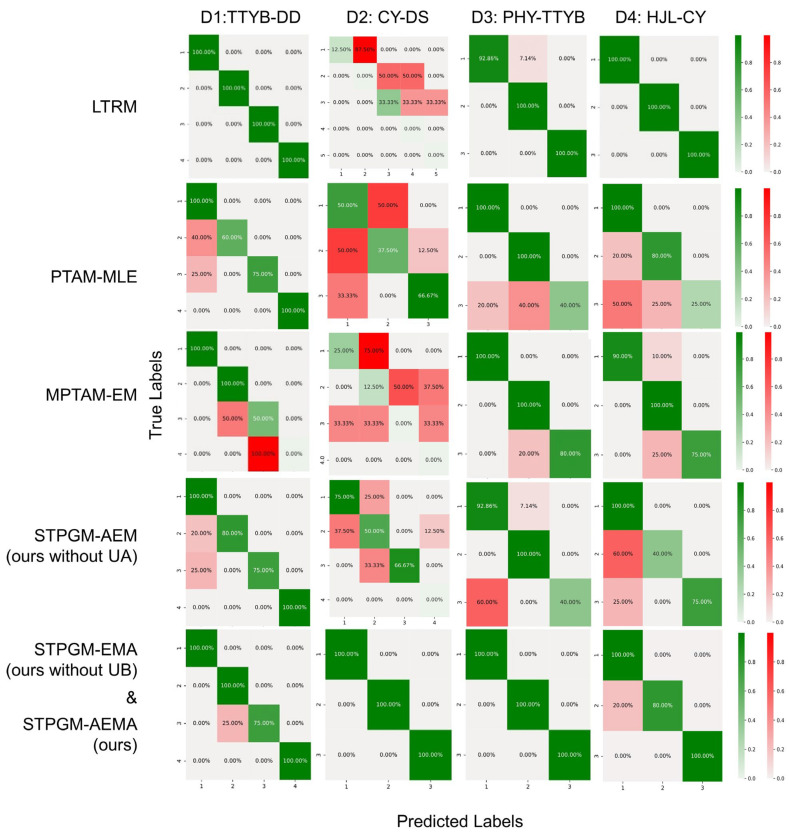
Comparison of the confusion matrix.

Figure 8 presents the classification results across four datasets (D1–D4) utilizing optimal parameters, depicted through a Confusion Matrix format. Each column is allocated to a dataset, while each row showcases the efficacy of a specific method applied to that dataset. Predicted labels are displayed along the horizontal axis, with true labels along the vertical axis. Areas of correct classification are marked in green, whereas inaccuracies are highlighted in red, accompanied by percentages that reflect the proportion of correct and incorrect classifications. The intensity of the color signifies proportionality, with darker shades indicating a higher frequency of occurrences. Owing to the consistent outcomes between the STPGM-EMA and STPGM-AEMA methods, their results have been consolidated for representation. 

The experimental analysis elucidates that while most algorithms fare well in scenarios with a single alternative train option, their efficacy diminishes in contexts with multiple train choices, illustrating a notable challenge in navigating complex classification landscapes. This delineates a direct linkage between the number of potential train choices and the escalation of uncertainty in passenger trajectories, inherently augmenting the likelihood of misclassification. A cross-comparison of various methods reveals that the UA module plays a crucial role in the STPGM-AEMA framework to capture data details. This highlights a direct correlation between the number of potential train selections and an escalation in passenger trajectory uncertainty.

Table 2 shows the results of the accuracy assessment of the different algorithms on each dataset. It is particularly noteworthy that STPGM-AEMA(ours) and STPGM-EMA (ours without UB) perform well on all datasets, while the other algorithms perform poorly at least on the D2 dataset. the prediction accuracy of the STPGM_AEMA method proposed in this article reaches more than 90% on all datasets, showing that the algorithm can cope with scenarios of different complexity levels. Despite some random errors, the overall robustness is good. 

The results are consistent with the hypothesis proposed in this paper that the complexity of the operating model and the station structure influence the accuracy of trajectory reconstruction. Specifically, in the D2 dataset, the destination station DS is an interchange and adopts a short-turning operation pattern during peak hours for commuting needs. Although DD is a transfer station, the operation mode of the D1 dataset is a simple mode. While the destination stations in the D3 and D4 datasets are non-transfer stations, their operation mode is also a simple mode. Besides, the passenger flow of the D3 dataset is the lowest. Therefore, the complexity of these four datasets, from high to low, is D2, D1, D3, and D4. The performance difference of D2 may be due to the operating modes of the origin station CY during peak hours; at the same time, the complex mode of the transfer destination station DS further increases the difficulty of prediction. This comparison reinforces the view that OD’s complexity of the scene directly affects the accuracy of the algorithm’s prediction of passenger trajectories.

From the average value, the STPGM-AEMA and STPGM-EMA algorithms have demonstrated exceptional performance, with all metrics exceeding 0.95, showcasing significantly superior classification capabilities compared to other methods. Following them are the LTRM and STPGM-AEM algorithms, which, despite performing well in certain scenarios, exhibit relatively lower overall stability, especially when faced with unevenly distributed dataset features or significant variability. In summary, the STPGM-AEMA approach presented in this study demonstrates exceptional performance across both macro and micro metrics, emphasizing the remarkable robustness of the proposed models. This outcome accentuates the precision of the STPGM-AEMA method devised in this research in processing intricate spatiotemporal data and accurately capturing passenger behavior patterns, underscoring its utility in complex urban rail transit analyses.

#### 5.4.2. Consistency Test Result 

It is evident that STPGM-AEMA and STPGM-EMA exhibit superior performance (as presented in Table 3), with K values exceeding 0.9. This observation suggests that as the sample size tends toward infinity, the estimator’s value can converge to the true parameter value. Subsequently, the LTRM algorithm demonstrates optimal performance on the D1 and D4 datasets but exhibits subpar results on the D2 dataset. It should be noted that for other algorithms, the stability of results may be significantly influenced by variations in walking distances and passenger paths at different entry stations within each dataset.

### 5.5. Results Interpretability Discussion

#### 5.5.1. Potential Train Sets Feature Analysis

**Figure 9:**
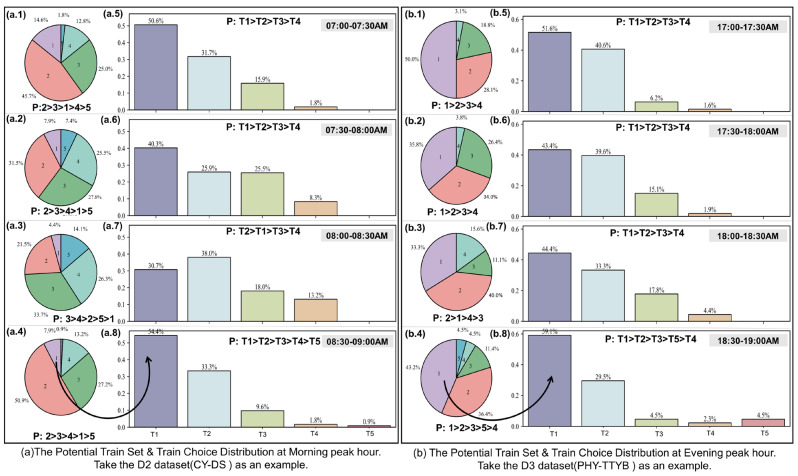
Comparison of typical OD on train selection and train selection distribution.

Figure 9 contrasts the distribution of potential train sets for typical OD pairs during peak hours (using datasets D2 and D3 as examples) with the distribution of train choices by passengers at the origin station. As shown in Figure 9(a.1–a.4) depict the distribution of potential train sets for passenger journeys entering the station in dataset D2 between 07:00 and 09:00 a.m. in half-hour increments, where 1–5 represent the number of train options and “P: >” indicates the statistical proportion ranking of train options. For instance, in Figure 9(a.1), “P: 2 > 3 > 1 > 4 > 5” indicates that the proportion of having two train options is the highest at 45.7%, followed by 3 (25%), 1 (14.6%), 4 (12.8%), and 5 (1.8%). Figure 9(b.1–b.4) follow a similar pattern. It is observed that during the morning peak, the statistical values for train options mostly range between 2 and 4, while during the evening peak, options of 1–2 are more prevalent, likely due to the higher frequency of train departures in the morning and relatively sparse intervals in the evening.

#### 5.5.2. Analysis of Latent Variable Z Distribution

Figure 9(a.5–a.8) illustrate the distribution of train choices at the origin station for all passenger journeys during the morning peak, in half-hour intervals within dataset D2, representing the distribution of the latent variable Z. T1–T5 denotes feasible train ID, with “P: >” indicating their statistical ranking based on chosen trains. For instance, in Figure 9(a.5), “P: T1 > T2 > T3 > T4” signifies that the first train has the highest selection proportion at 50.6%, followed by T2 (31.7%), T3 (15.9%), and T4 (1.8%). A similar pattern is observed in Figure 9(b.5–b.8). It is evident that during the morning peak, there are variations in train choice probabilities across different time slots; however, a more consistent exponential distribution is apparent during the evening peak hours.

Interestingly, during the evening peak, there is a clear alignment between the ranking of potential train numbers and the sequence of chosen train ID, which is not as evident in the morning peak. For instance, between 08:30 and 09:00 a.m., despite only 7.9% of choices having one train option available, the proportion of selecting the first train reaches 54.4%. In contrast, during the evening peak, when only 43.2% of choices have one train option available, the selection proportion for the first train rises to 59.1%. This discrepancy can be attributed to the higher demand for comfort among evening peak passengers, who prioritize seating and exhibit a slower walking speed compared to morning commuters. In contrast, morning commuters prioritize quick arrival and tend to adopt a “board if possible” behavior.

#### 5.5.3. Analysis of the Changing Process of Attention Mechanism

**Figure 10:**
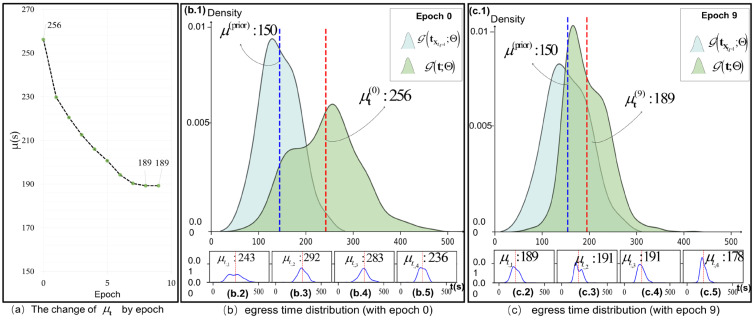
Q value and PDF distribution changes.

Figure 10a depicts the variation of value across iterations, with the horizontal axis representing the number of iterations and the vertical axis indicating changes in value μ and pdf of the latent variable t, taking the D2 data set as an example (unit in second). Figure 10b,c display PDF distributions of the initial 0 and final iteration 9, respectively. The pdf distribution based on $t_{X_{L_{I}=1}}$ is represented by a blue-filled curve, while that based on t is depicted with a green-filled curve. The blue vertical line represents the acquired prior value ($\mu^{\left(\mathrm{prior}\right)}=150s$), serving as a reference for parameter variation, whereas the red vertical line indicates the current iteration round’s value μ. It can be observed that the prior value exhibits left-skewness, which decreases from $u^{\left(0\right)}=256s$ to $u^{\left(9\right)}=189s$, after learning through UA module and stabilizes thereafter. Notably, Figure 10(b.2–b.5) and 10(c.2–c.5) demonstrate different PDF distribution shapes of vectors at various position indexes (from left to right: T1–T4), corresponding to the first and last rounds, respectively. Evidently, as iterations increase, they tend to align more closely with $u^{\left(9\right)}$.

Experimental results demonstrate that the variables of the key-value pairs (K,V) exhibit a tendency to align more closely with the matrix q, indicating that the UA module enables interactive learning of both passenger egress time $t_{i,j}$ and destination station time distribution $G\left(t;\Theta \right)$. 

#### 5.5.4. Individual Trajectory Visualization

**Figure 11:**
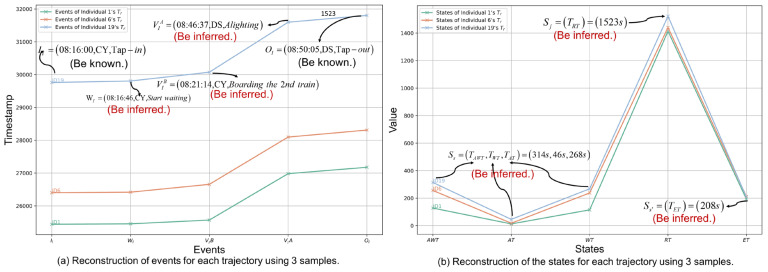
Individuals’ reconstructed trajectories visualization.

The events and state values involved in the reconstructed trajectory are visualized using 3 samples of the D2 dataset, as shown in Figure 11a and Figure 11b, respectively. Figure 11a shows the reconstructed trajectory of individual “ID19”, where the tap-in and tap-out events are known and marked as “be known” in black font. Other events are inferred and marked “be inferred” in red font. It can be seen that passenger “ID19” has already inferred that he boarded train 2 at station CY at 08:21:14, and the interpretation of other event information is similar. Of course, if the trajectories of all passengers are displayed, the congestion and distribution of passengers waiting on the platform can be further analyzed, this is not the focus of this article. Figure 11b shows the inferred state information of each individual. It can be seen that the egress times are indeed relatively similar, which in turn confirms the effectiveness of the model STPGM proposed in this article. Furthermore, detailed error analysis is discussed in depth in the next section.

#### 5.5.5. Residual Analysis of Trajectory Reconstruction Fragments

The reconstruction error of the involved ITT data, encompassing temporal attributes $T_{E_{h}}$ and state characteristics $S_{f}$ of the event, is assessed based on residuals $err(\cdot )=tr_{I}-\hat{tr_{I}}$ (unit in second). Further, err(·)>0 indicates predicted occurrence times earlier than the actual events, while positive residuals suggest later predictions. Regarding state values, err(·)>0 denote underpredictions, whereas positive values indicate overpredictions. 

**Figure 12:**
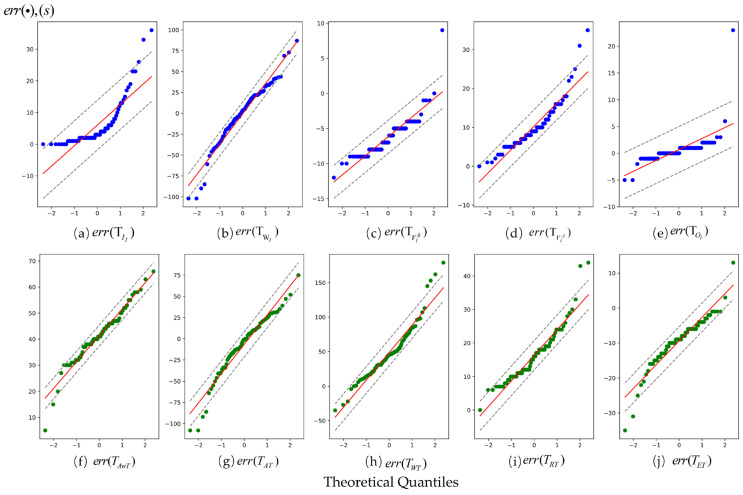
Event and state residuals Q-Q plots of trajectory fragments.

Figure 12 employs Q-Q plots to demonstrate the normality of each event-time variable. The horizontal axis represents theoretical quantiles of the probability distribution, and the vertical axis reflects percentiles of residual values. The red line represents the regression line satisfying either $T_{E_{h}}=\hat{T_{E_{h}}}$ (in Figure 12a–e) or $S_{f}=\hat{S_{f}}$ (in Figure 12f–j). Two grey dashed lines indicate a 95% confidence interval, with individual residual err(·) denoted by points on the plot. 

The analysis in Figure 12 reveals that, when considering the deviation from events, it is evident that, apart from $err(T_{I_{I}})$ being influenced by system errors, deviations in $T_{V_{I}^{B}}$ and $T_{V_{I}^{A}}$ occur due to disregarding the time spent onboarding and alighting. To enhance the model, future research can incorporate a deviation correction coefficient. Notably, the largest deviation value about $T_{W_{I}}$ primarily stems from insufficient observational information and significant randomness. Regarding state values, aside from errors $err(T_{RT})$ resulting from individual heterogeneity’s perception bias, most values are predominantly positive due to their association with calculation methods. It is noteworthy that the error value $err(T_{ET})$ is minimal, thus confirming the efficacy of our proposed method. Furthermore, except for $err(T_{I_{I}})$, a majority of data points fall within an interval range while exhibiting residuals close to normality, validating the effectiveness of the ITR method proposed in this paper. The Q-Q plots effectively visualize the normality or deviation of residuals, offering a statistical basis to assess the model’s performance in reconstructing travel event timelines.

## 6. Conclusions

In this paper, an automatic inference method for ITR has been proposed, namely STPGM-AEMA, which aims to infer missing information from incomplete information. The method effectively recovers rich semantic and state information about each individual trajectory using only AFC and AVL data. A GOLN rule is introduced in the model as a bridge from observed data to inferred information. On this basis, an information interaction representation module for global and local latent variables was designed, which effectively promotes autonomous communication of information between individuals and the system, eliminating dependence on manual survey data. Secondly, the proposed parameter learning algorithm AEMA enhances the EM algorithm by adaptively introducing a priori parameters and a key-value attention mechanism. It not only improves the stability and convergence speed of parameters but also automatically samples the walking time of individual and egress time distributions to deal with missing data problems. In addition, combined with ITT data, three methods and two ablation experimental methods were comparatively analyzed. The results show that the proposed STPGM-AEMA method performs well in terms of accuracy and robustness, and the accuracy can reach 0.95 (95%), which is at least 15% more accurate than the traditional methods (i.e., PTAM-MLE and MPTAM-EM). 

It is worth noting that interpretability analysis was performed on key parts of the STPGM-AEMA method, including potential set feature mining analysis, latent variable distribution analysis, the role of the attention mechanism, and temporal residual analysis. On this basis, some possible directions for improvement could be as follows: (1) addressing the limitations of the proposed model in estimating individual trajectories between OD pairs with insufficient data, as any lack of prior information will adversely affect the utility of the UB module; (2) Currently, a simple normalization function is used in the UA module. In future research, the application of other activation functions (Leaky ReLU, weighted Softmax, etc.) in multi-class imbalance problems can be explored to enhance model fitting capabilities; (3) Extend the model formulation to include route choice probability, passenger type, station type, or operation strategies as additional model parameters; and (4) Although the AEMA algorithm proposed in this paper employs offline training, the average training time for a single dataset in this study is approximately 15.79 s, which adequately satisfies the requirements for fast trajectory reconstruction of complete samples within a single OD pair. Future work can explore the possibility of integrating real-time sample generation and correction modules to achieve real-time personal travel trajectory prediction. Certainly, this requires significant extensions to existing models.

## Figures and Tables

**Table 2:** Results of accuracy evaluation metrics.

Dataset	Methods	$P_{Macro}$	$R_{Macro}$	${F1}_{Macro}$	$P_{Micro}$	$R_{Micro}$	${F1}_{Micro}$
D1: TTYB-DD	LTRM	**1.00 × 10^0 1^**	**1.00 × 10^0^**	**1.00 × 10^0^**	**1.00 × 10^0^**	**1.00 × 10^0^**	**1.00 × 10^0^**
	PTAM-MLE	9.46 × 10^−1^	8.38 × 10^−1^	8.72 × 10^−1^	8.57 × 10^−1^	8.57 × 10^−1^	8.57 × 10^−1^
	MPTAM-EM	5.95 × 10^−1^	6.25 × 10^−1^	6.01 × 10^−1^	8.57 × 10^−1^	8.57 × 10^−1^	8.57 × 10^−1^
	STPGM-AEM	9.62 × 10^−1 1^	8.88 × 10^−1^	9.16 × 10^−1^	9.05 × 10^−1^	9.05 × 10^−1^	9.05 × 10^−1^
	STPGM-EMA	9.58 × 10^−1^	9.38 × 10^−1^	9.42 × 10^−1^	9.52 × 10^−1^	9.52 × 10^−1^	9.52 × 10^−1^
	STPGM-AEMA(ours)	9.58 × 10^−1^	9.38 × 10^−1^	9.42 × 10^−1^	9.52 × 10^−1^	9.52 × 10^−1^	9.52 × 10^−1^
D2: CY-DS	LTRM	2.40 × 10^−1^	9.17 × 10^−2^	9.44 × 10^−2^	1.05 × 10^−1^	1.05 × 10^−1^	1.05 × 10^−1^
	PTAM-MLE	5.13 × 10^−1^	5.14 × 10^−1^	5.12 × 10^−1^	4.74 × 10^−1^	4.74 × 10^−1^	4.74 × 10^−1^
	MPTAM-EM	1.98 × 10^−1^	9.38 × 10^−2^	1.22 × 10^−1^	1.58 × 10^−1^	1.58 × 10^−1^	1.58 × 10^−1^
	STPGM-AEM	5.60 × 10^−1^	4.79 × 10^−1^	5.10 × 10^−1^	6.32 × 10^−1^	6.32 × 10^−1^	6.32 × 10^−1^
	STPGM-EMA	**1.00 × 10^0^**	**1.00 × 10^0^**	**1.00 × 10^0^**	**1.00 × 10^0^**	**1.00 × 10^0^**	**1.00 × 10^0^**
	STPGM-AEMA(ours)	**1.00 × 10^0^**	**1.00 × 10^0^**	**1.00 × 10^0^**	**1.00 × 10^0^**	**1.00 × 10^0^**	**1.00 × 10^0^**
D3: PHY-TTYB	LTRM	8.33 × 10^−1^	9.76 × 10^−1^	8.77 × 10^−1^	9.50 × 10^−1^	9.50 × 10^−1^	9.50 × 10^−1^
	PTAM-MLE	7.56 × 10^−1^	8.00 × 10^−1^	6.79 × 10^−1^	8.50 × 10^−1^	8.50 × 10^−1^	8.50 × 10^−1^
	MPTAM-EM	8.33 × 10^−1^	9.33 × 10^−1^	8.52 × 10^−1^	9.50 × 10^−1^	9.50 × 10^−1^	9.50 × 10^−1^
	STPGM-AEM	7.71 × 10^−1^	7.76 × 10^−1^	7.02 × 10^−1^	8.00 × 10^−1^	8.00 × 10^−1^	8.00 × 10^−1^
	STPGM-EMA	**1.00 × 10^0^**	**1.00 × 10^0^**	**1.00 × 10^0^**	**1.00 × 10^0^**	**1.00 × 10^0^**	**1.00 × 10^0^**
	STPGM-AEMA(ours)	**1.00 × 10^0^**	**1.00 × 10^0^**	**1.00 × 10^0^**	**1.00 × 10^0^**	**1.00 × 10^0^**	**1.00 × 10^0^**
D4: HJL-CY	LTRM	**1.00 × 10^0^**	**1.00 × 10^0^**	**1.00 × 10^0^**	**1.00 × 10^0^**	**1.00 × 10^0^**	**1.00 × 10^0^**
	PTAM-MLE	8.56 × 10^−1^	6.83 × 10^−1^	6.90 × 10^−1^	7.89 × 10^−1^	7.89 × 10^−1^	7.89 × 10^−1^
	MPTAM-EM	9.05 × 10^−1^	8.83 × 10^−1^	8.79 × 10^−1^	8.95 × 10^−1^	8.95 × 10^−1^	8.95 × 10^−1^
	STPGM-AEM	9.05 × 10^−1^	7.17 × 10^−1^	7.54 × 10^−1^	7.89 × 10^−1^	7.89 × 10^−1^	7.89 × 10^−1^
	STPGM-EMA	9.67 × 10^−1^	9.33 × 10^−1^	9.45 × 10^−1^	9.44 × 10^−1^	9.44 × 10^−1^	9.44 × 10^−1^
	STPGM-AEMA(ours)	9.67 × 10^−1^	9.33 × 10^−1^	9.45 × 10^−1^	9.44 × 10^−1^	9.44 × 10^−1^	9.44 × 10^−1^
Average	LTRM	7.54 × 10^−1^	7.59 × 10^−1^	7.31 × 10^−1^	7.51 × 10^−1^	7.51 × 10^−1^	7.51 × 10^−1^
	PTAM-MLE	7.68 × 10^−1^	7.09 × 10^−1^	6.88 × 10^−1^	7.43 × 10^−1^	7.43 × 10^−1^	7.43 × 10^−1^
	MPTAM-EM	6.33 × 10^−1^	6.34 × 10^−1^	6.14 × 10^−1^	7.15 × 10^−1^	7.15 × 10^−1^	7.15 × 10^−1^
	STPGM-AEM	7.99 × 10^−1^	7.15 × 10^−1^	7.20 × 10^−1^	7.81 × 10^−1^	7.81 × 10^−1^	7.81 × 10^−1^
	STPGM-EMA	**9.81 × 10^−1^**	**9.68 × 10^−1^**	**9.72 × 10^−1^**	**9.74 × 10^−1^**	**9.74 × 10^−1^**	**9.74 × 10^−1^**
	STPGM-AEMA(ours)	**9.81 × 10^−1^**	**9.68 × 10^−1^**	**9.72 × 10^−1^**	**9.74 × 10^−1^**	**9.74 × 10^−1^**	**9.74 × 10^−1^**

^1^ Bold denotes the best result, and underline denotes the second-best result. The same is below in Table 3.

**Table 3:** Results of Cohen’s Kappa consistency test.

Method	D1: TTYB-DD	D2: CY-DS	D3: PHY-TTYB	D4: HJL-CY	Average
LTRM	**1.00 × 10^0^**	−1.45 × 10^−1^	8.95 × 10^−1^	**1.00 × 10^0^**	6.87 × 10^−1^
PTAM-MLE	7.57 × 10^−1^	1.52 × 10^−1^	6.61 × 10^−1^	6.24 × 10^−1^	5.48 × 10^−1^
MPTAM-EM	7.69 × 10^−1^	−1.65 × 10^−1^	8.90 × 10^−1^	8.30 × 10^−1^	5.81 × 10^−1^
STPGM-AEM	8.42 × 10^−1^	4.14 × 10^−1^	5.12 × 10^−1^	6.18 × 10^−1^	5.96 × 10^−1^
STPGM-EMA	9.24 × 10^−1^	**1.00 × 10^0^**	**1.00 × 10^0^**	9.09 × 10^−1^	**9.58 × 10^−1^**
STPGM-AEMA(ours)	9.24 × 10^−1^	**1.00 × 10^0^**	**1.00 × 10^0^**	9.09 × 10^−1^	**9.58 × 10^−1^**

## Data Availability

Data are contained within the article.

## Appendix A. Notations and Definitions

**Symbol**	**Definition**
i	Passenger index.
s, $s^{\prime }$	The origin and destination stations of an individual trip, respectively.
t, $t^{\prime }$	The tap-in and tap-out time of an individual trip index, respectively.
$I_{\left(t,s)\to (t^{\prime },s^{\prime }\right)}^{i}$	Itinerary index, where $\left(t,s)\to (t^{\prime },s^{\prime }\right)$ indicates that passenger i enter the origin station s at t and leave the destination station $s^{\prime }$ at $t^{\prime }$, abbreviated as I.
$I_{s,t}$, $I_{s^{\prime },t^{\prime }}$	The tap-in and tap-out time of an individual itinerary.
tj	Train index of all trains between OD pairs.
$tj_{I,id}$	A unique symbol representing the ID of train in journal I.
$tj_{s,at}$, $tj_{s,dt}$, $tj_{s^{\prime },at}$, $tj_{s^{\prime },dt}$	The arrival time and departure time at origin station s/destination station $s^{\prime }$ of the train tj, respectively.
$tj_{\mathrm{fs},q}$, $tj_{s,q}$, $tj_{s^{\prime },q}$, $tj_{\mathrm{es},q}$	The order number of the train tj at first station fs, station s, station $s^{\prime }$ and last station es.
$J_{I^{\left(t,s\right)}}$, $J_{I^{\left(t^{\prime },s^{\prime }\right)}}$	The set of feasible train choices at origin station s/destination station $s^{\prime }$ for an itinerary I, respectively.
$J_{I}$	The set of feasible train choices for an itinerary I, denoted as {1,2,⋯,j}, with the index being j, $L_{I}$ represents the ordered sequence of train options available. The total length of this sequence is, with the dimension being $1\times L_{I}$.
$T_{i^{I(s^{\prime })}}^{\mathrm{eg}}$	The potential set of egress time for an itinerary I, denoted as $\left\{t_{i,1},t_{i,2},\cdots ,t_{i,j}\right\}$, abbreviated as $T_{I}^{\mathrm{eg}}$.
$t_{i,j}$	The jth potential egress time value for passenger i in itinerary I.

## Appendix B. The Recorded Data from the Trajectory Simulation Experiment

Date	2023/3/21				
**PID**	15****36				
**Itinerary index**	234				
**L1**	**S1**	**Tap-in Time**	**To platform**	**Boarding Time**	**Train Departure Time**
6	623	17:34:50	17:36:55	17:38:40	17:38:50
**L2**	**S2**	**Train Arrival Time**	**Alighting Time**	**Tap-out time**	
6	633	17:58:56	17:59:05	17:59:59	

## Appendix C. The Table Presents the States of the Trajectory Calculation Results

Date	2023/3/21				
**PID**	15****36				
**Itinerary index**	234				
**OD pair**	**Access time(s)**	**Train ID**	**Waiting time(s)**	**Riding time(s)**	**Egress time(s)**
623-633	125	1222	105	1206	54
